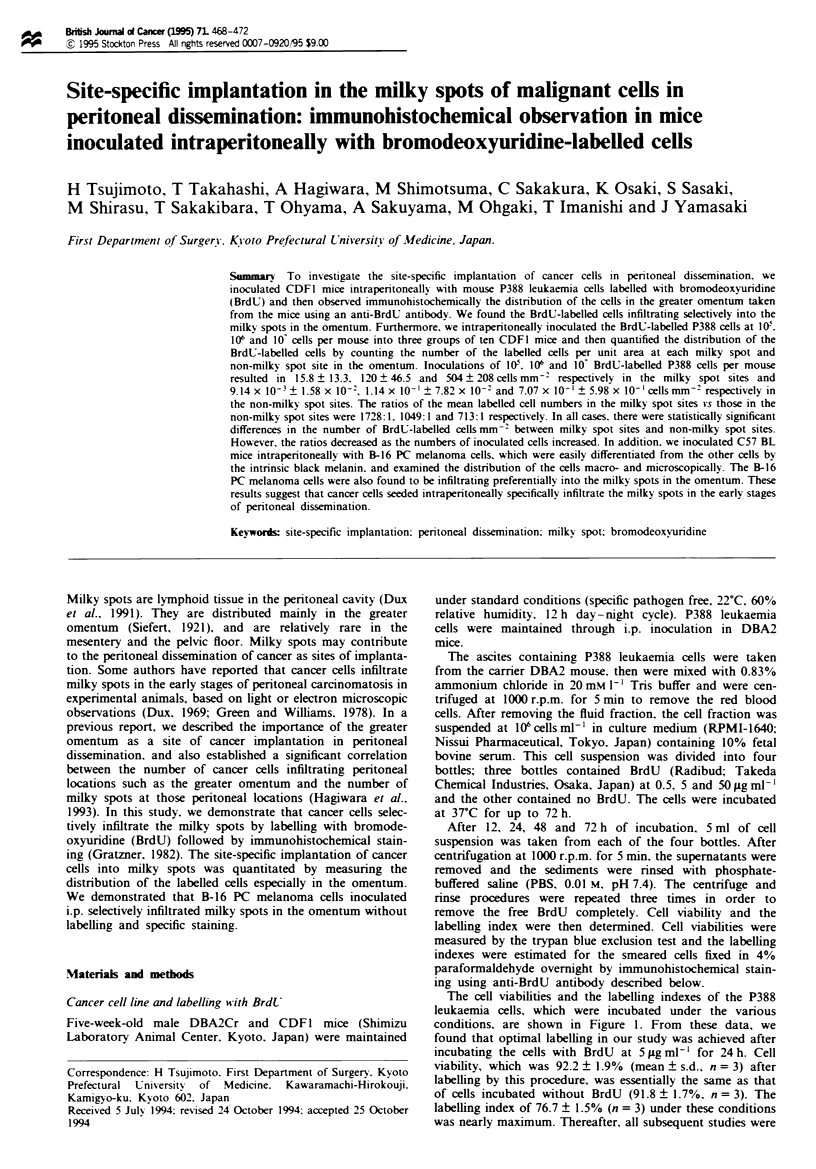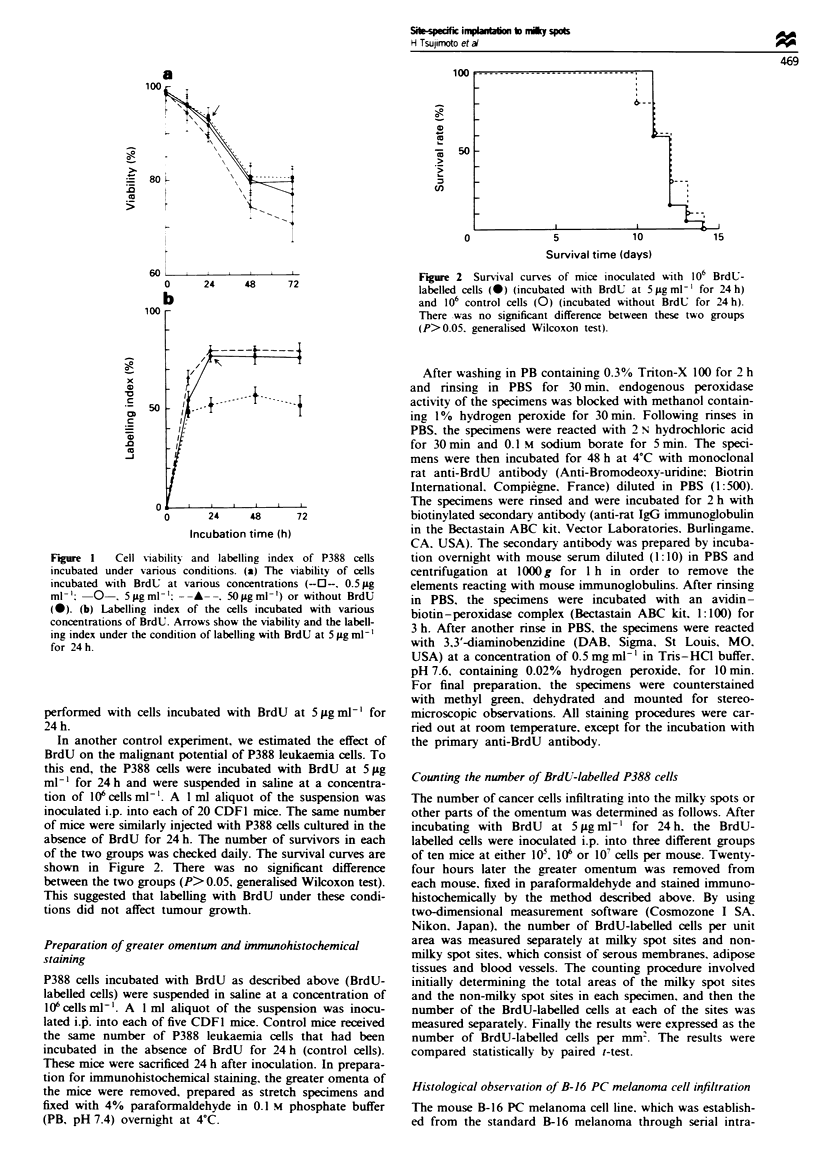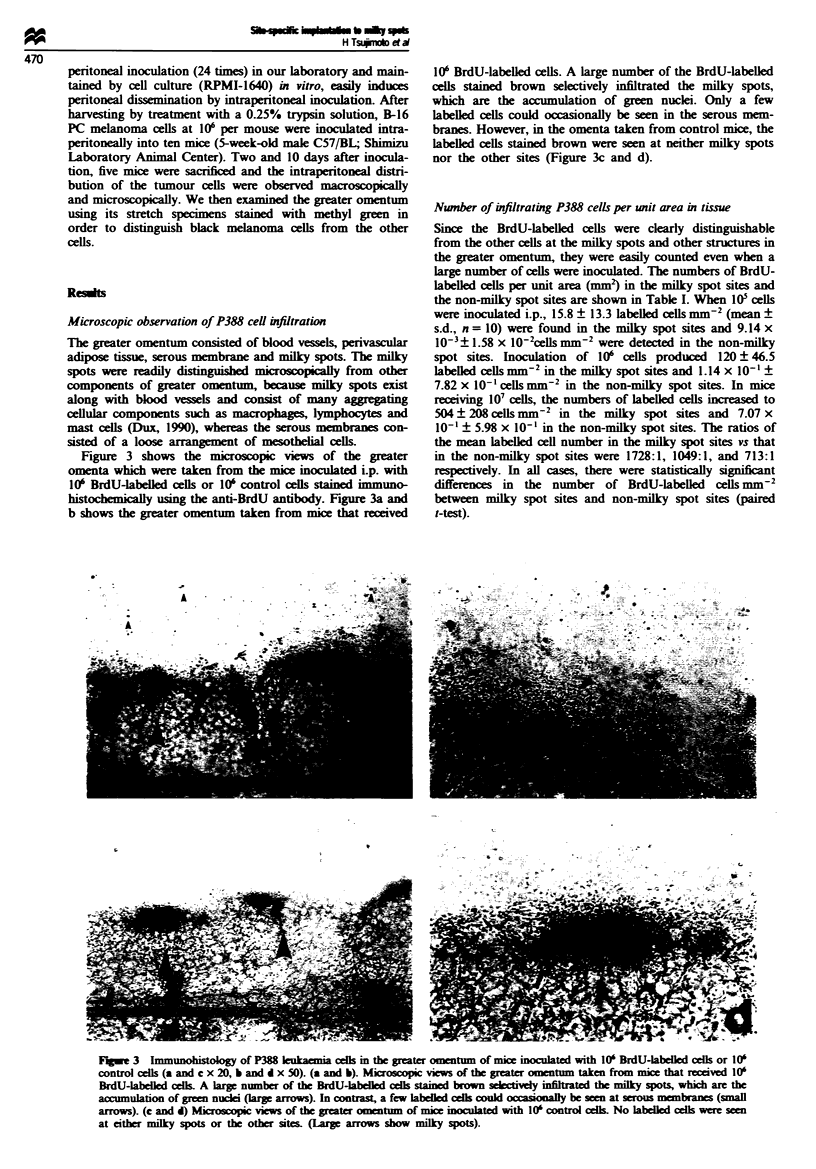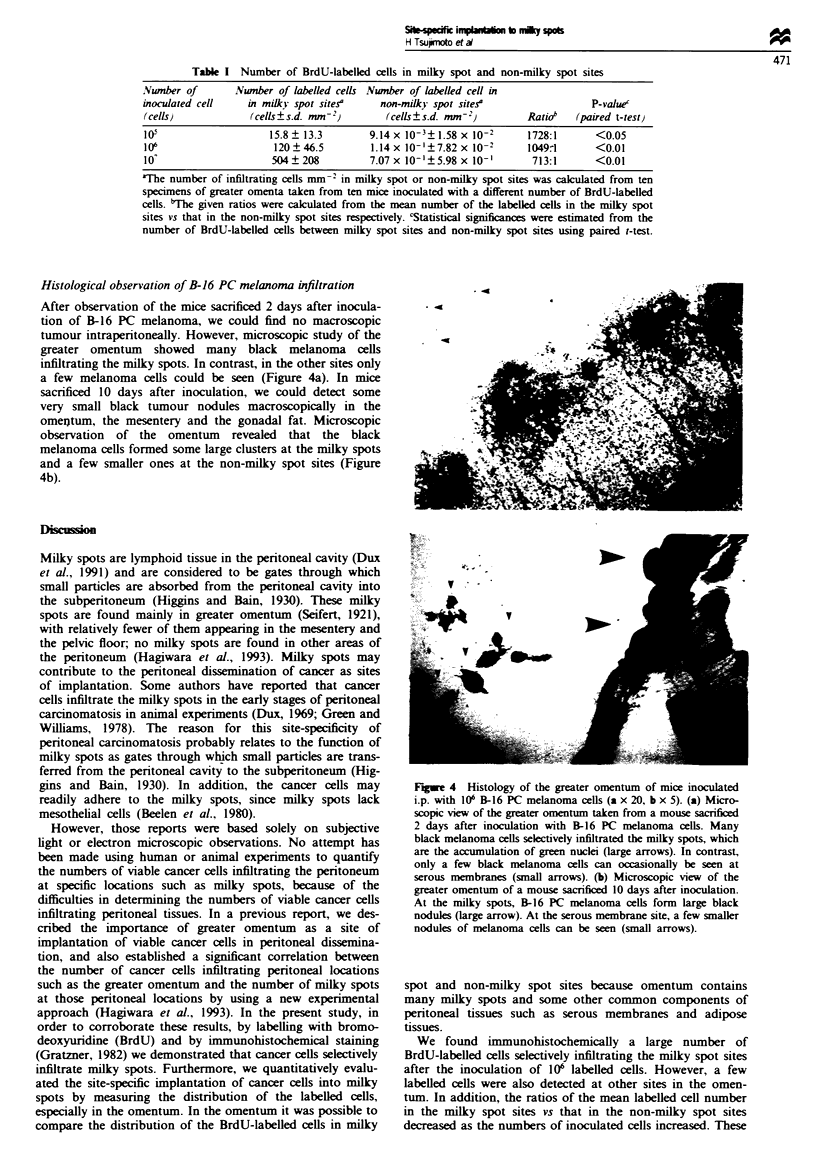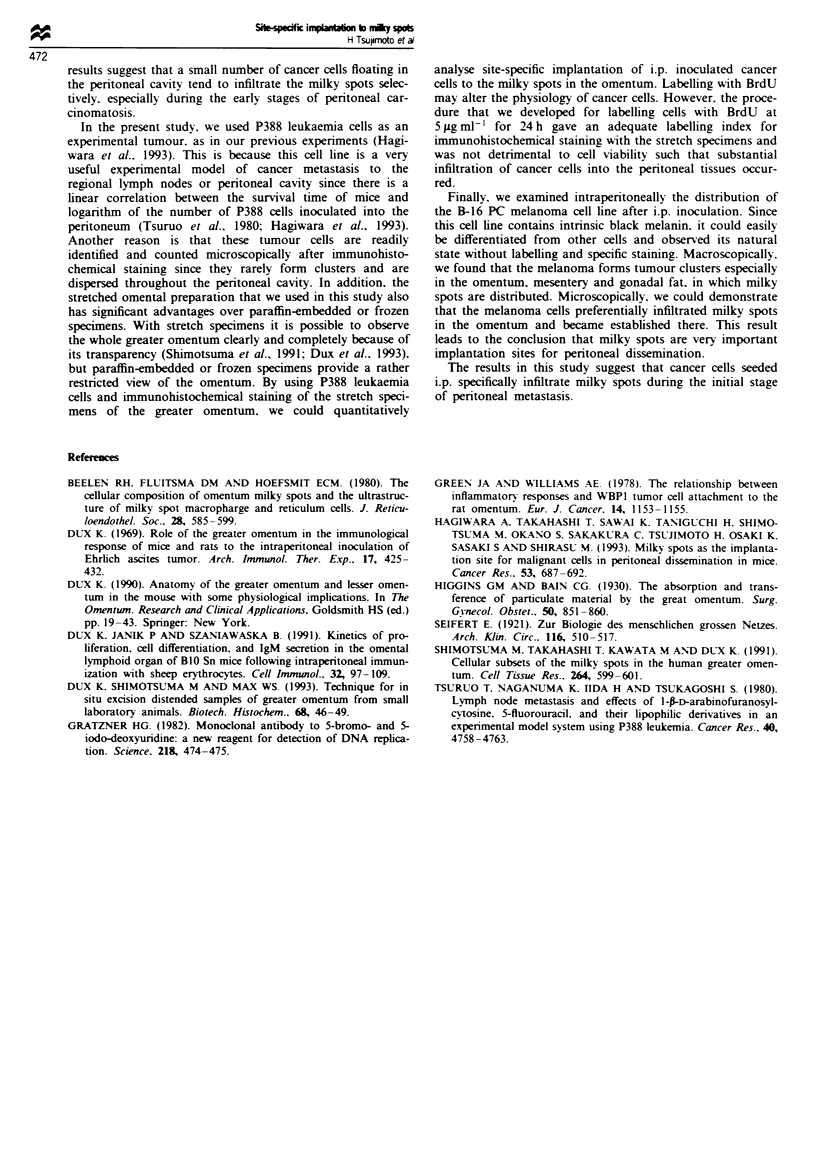# Site-specific implantation in the milky spots of malignant cells in peritoneal dissemination: immunohistochemical observation in mice inoculated intraperitoneally with bromodeoxyuridine-labelled cells.

**DOI:** 10.1038/bjc.1995.95

**Published:** 1995-03

**Authors:** H. Tsujimoto, T. Takhashi, A. Hagiwara, M. Shimotsuma, C. Sakakura, K. Osaki, S. Sasaki, M. Shirasu, T. Sakakibara, T. Ohyama

**Affiliations:** First Department of Surgery, Kyoto Prefectural University of Medicine, Japan.

## Abstract

**Images:**


British Journal of Cancer (1995) 71, 468-472

© 1995 Stockton Press All rights reserved 0007-0920/95 $9.00

### Site-specific implantation in the milky spots of malignant cells in peritoneal dissemination: immunohistochemical observation in mice inoculated intraperitoneally with bromodeoxyuridine-labelled cells

H Tsujimoto, T Takahashi, A Hagiwara, M Shimotsuma, C Sakakura, K Osaki, S Sasaki, M Shirasu, T Sakakibara, T Ohyama, A Sakuyama, M Ohgaki, T Imanishi and J Yamasaki

*First Department of Surgery, Kyoto Prefectural University of Medicine, Japan.*

**Summary** To investigate the site-specific implantation of cancer cells in peritoneal dissemination, we inoculated CDF1 mice intraperitoneally with mouse P388 leukaemia cells labelled with bromodeoxyuridine (BrdU) and then observed immunohistochemically the distribution of the cells in the greater omentum taken from the mice using an anti-BrdU antibody. We found the BrdU-labelled cells infiltrating selectively into the milky spots in the omentum. Furthermore, we intraperitoneally inoculated the BrdU-labelled P388 cells at $10^5$, $10^6$ and $10^7$ cells per mouse into three groups of ten CDF1 mice and then quantified the distribution of the BrdU-labelled cells by counting the number of the labelled cells per unit area at each milky spot and non-milky spot site in the omentum. Inoculations of $10^5$, $10^6$ and $10^7$ BrdU-labelled P388 cells per mouse resulted in $15.8 \pm 13.3$, $120 \pm 46.5$ and $504 \pm 208$ cells mm$^{-2}$ respectively in the milky spot sites and $9.14 \times 10^{-3} \pm 1.58 \times 10^{-2}$, $1.14 \times 10^{-1} \pm 7.82 \times 10^{-2}$ and $7.07 \times 10^{-1} \pm 5.98 \times 10^{-1}$ cells mm$^{-2}$ respectively in the non-milky spot sites. The ratios of the mean labelled cell numbers in the milky spot sites *vs* those in the non-milky spot sites were 1728:1, 1049:1 and 713:1 respectively. In all cases, there were statistically significant differences in the number of BrdU-labelled cells mm$^{-2}$ between milky spot sites and non-milky spot sites. However, the ratios decreased as the numbers of inoculated cells increased. In addition, we inoculated C57 BL mice intraperitoneally with B-16 PC melanoma cells, which were easily differentiated from the other cells by the intrinsic black melanin, and examined the distribution of the cells macro- and microscopically. The B-16 PC melanoma cells were also found to be infiltrating preferentially into the milky spots in the omentum. These results suggest that cancer cells seeded intraperitoneally specifically infiltrate the milky spots in the early stages of peritoneal dissemination.

**Keywords:** site-specific implantation; peritoneal dissemination; milky spot; bromodeoxyuridine

Milky spots are lymphoid tissue in the peritoneal cavity (Dux *et al.*, 1991). They are distributed mainly in the greater omentum (Siefert, 1921), and are relatively rare in the mesentery and the pelvic floor. Milky spots may contribute to the peritoneal dissemination of cancer as sites of implantation. Some authors have reported that cancer cells infiltrate milky spots in the early stages of peritoneal carcinomatosis in experimental animals, based on light or electron microscopic observations (Dux, 1969; Green and Williams, 1978). In a previous report, we described the importance of the greater omentum as a site of cancer implantation in peritoneal dissemination, and also established a significant correlation between the number of cancer cells infiltrating peritoneal locations such as the greater omentum and the number of milky spots at those peritoneal locations (Hagiwara *et al.*, 1993). In this study, we demonstrate that cancer cells selectively infiltrate the milky spots by labelling with bromodeoxyuridine (BrdU) followed by immunohistochemical staining (Gratzner, 1982). The site-specific implantation of cancer cells into milky spots was quantitated by measuring the distribution of the labelled cells especially in the omentum. We demonstrated that B-16 PC melanoma cells inoculated i.p. selectively infiltrated milky spots in the omentum without labelling and specific staining.

### Materials and methods

### *Cancer cell line and labelling with BrdU*

Five-week-old male DBA2Cr and CDF1 mice (Shimizu Laboratory Animal Center, Kyoto, Japan) were maintained under standard conditions (specific pathogen free, 22°C, 60% relative humidity, 12 h day-night cycle). P388 leukaemia cells were maintained through i.p. inoculation in DBA2 mice.

The ascites containing P388 leukaemia cells were taken from the carrier DBA2 mouse, then were mixed with 0.83% ammonium chloride in 20 mM l$^{-1}$ Tris buffer and were centrifuged at 1000 r.p.m. for 5 min to remove the red blood cells. After removing the fluid fraction, the cell fraction was suspended at $10^6$ cells ml$^{-1}$ in culture medium (RPMI-1640; Nissui Pharmaceutical, Tokyo, Japan) containing 10% fetal bovine serum. This cell suspension was divided into four bottles; three bottles contained BrdU (Radibud; Takeda Chemical Industries, Osaka, Japan) at 0.5, 5 and 50 $\mu$g ml$^{-1}$ and the other contained no BrdU. The cells were incubated at 37°C for up to 72 h.

After 12, 24, 48 and 72 h of incubation, 5 ml of cell suspension was taken from each of the four bottles. After centrifugation at 1000 r.p.m. for 5 min, the supernatants were removed and the sediments were rinsed with phosphate-buffered saline (PBS, 0.01 M, pH 7.4). The centrifuge and rinse procedures were repeated three times in order to remove the free BrdU completely. Cell viability and the labelling index were then determined. Cell viabilities were measured by the trypan blue exclusion test and the labelling indexes were estimated for the smeared cells fixed in 4% paraformaldehyde overnight by immunohistochemical staining using anti-BrdU antibody described below.

The cell viabilities and the labelling indexes of the P388 leukaemia cells, which were incubated under the various conditions, are shown in Figure 1. From these data, we found that optimal labelling in our study was achieved after incubating the cells with BrdU at 5 $\mu$g ml$^{-1}$ for 24 h. Cell viability, which was $92.2 \pm 1.9$% (mean ± s.d., $n = 3$) after labelling by this procedure, was essentially the same as that of cells incubated without BrdU ($91.8 \pm 1.7$%, $n = 3$). The labelling index of $76.7 \pm 1.5$% ($n = 3$) under these conditions was nearly maximum. Thereafter, all subsequent studies were

Correspondence: H Tsujimoto, First Department of Surgery, Kyoto Prefectural University of Medicine, Kawaramachi-Hirokouji, Kamigyo-ku, Kyoto 602, Japan
Received 5 July 1994; revised 24 October 1994; accepted 25 October 1994

**Figure 1** Cell viability and labelling index of P388 cells incubated under various conditions. (**a**) The viability of cells incubated with BrdU at various concentrations (--□--, 0.5 μg ml$^{-1}$; —○—, 5 μg ml$^{-1}$; --▲--, 50 μg ml$^{-1}$) or without BrdU (●). (**b**) Labelling index of the cells incubated with various concentrations of BrdU. Arrows show the viability and the labelling index under the condition of labelling with BrdU at 5 μg ml$^{-1}$ for 24 h.

performed with cells incubated with BrdU at 5 μg ml$^{-1}$ for 24 h.

In another control experiment, we estimated the effect of BrdU on the malignant potential of P388 leukaemia cells. To this end, the P388 cells were incubated with BrdU at 5 μg ml$^{-1}$ for 24 h and were suspended in saline at a concentration of $10^6$ cells ml$^{-1}$. A 1 ml aliquot of the suspension was inoculated i.p. into each of 20 CDF1 mice. The same number of mice were similarly injected with P388 cells cultured in the absence of BrdU for 24 h. The number of survivors in each of the two groups was checked daily. The survival curves are shown in Figure 2. There was no significant difference between the two groups ($P > 0.05$, generalised Wilcoxon test). This suggested that labelling with BrdU under these conditions did not affect tumour growth.

### *Preparation of greater omentum and immunohistochemical staining*

P388 cells incubated with BrdU as described above (BrdU-labelled cells) were suspended in saline at a concentration of $10^6$ cells ml$^{-1}$. A 1 ml aliquot of the suspension was inoculated i.p. into each of five CDF1 mice. Control mice received the same number of P388 leukaemia cells that had been incubated in the absence of BrdU for 24 h (control cells). These mice were sacrificed 24 h after inoculation. In preparation for immunohistochemical staining, the greater omenta of the mice were removed, prepared as stretch specimens and fixed with 4% paraformaldehyde in 0.1 M phosphate buffer (PB, pH 7.4) overnight at 4°C.

**Figure 2** Survival curves of mice inoculated with $10^6$ BrdU-labelled cells (●) (incubated with BrdU at 5 μg ml$^{-1}$ for 24 h) and $10^6$ control cells (○) (incubated without BrdU for 24 h). There was no significant difference between these two groups ($P > 0.05$, generalised Wilcoxon test).

After washing in PB containing 0.3% Triton-X 100 for 2 h and rinsing in PBS for 30 min, endogenous peroxidase activity of the specimens was blocked with methanol containing 1% hydrogen peroxide for 30 min. Following rinses in PBS, the specimens were reacted with 2 N hydrochloric acid for 30 min and 0.1 M sodium borate for 5 min. The specimens were then incubated for 48 h at 4°C with monoclonal rat anti-BrdU antibody (Anti-Bromodeoxy-uridine; Biotrin International, Compiègne, France) diluted in PBS (1:500). The specimens were rinsed and were incubated for 2 h with biotinylated secondary antibody (anti-rat IgG immunoglobulin in the Bectastain ABC kit, Vector Laboratories, Burlingame, CA, USA). The secondary antibody was prepared by incubation overnight with mouse serum diluted (1:10) in PBS and centrifugation at 1000 *g* for 1 h in order to remove the elements reacting with mouse immunoglobulins. After rinsing in PBS, the specimens were incubated with an avidin–biotin–peroxidase complex (Bectastain ABC kit, 1:100) for 3 h. After another rinse in PBS, the specimens were reacted with 3,3′-diaminobenzidine (DAB, Sigma, St Louis, MO, USA) at a concentration of 0.5 mg ml$^{-1}$ in Tris–HCl buffer, pH 7.6, containing 0.02% hydrogen peroxide, for 10 min. For final preparation, the specimens were counterstained with methyl green, dehydrated and mounted for stereomicroscopic observations. All staining procedures were carried out at room temperature, except for the incubation with the primary anti-BrdU antibody.

### *Counting the number of BrdU-labelled P388 cells*

The number of cancer cells infiltrating into the milky spots or other parts of the omentum was determined as follows. After incubating with BrdU at 5 μg ml$^{-1}$ for 24 h, the BrdU-labelled cells were inoculated i.p. into three different groups of ten mice at either $10^5$, $10^6$ or $10^7$ cells per mouse. Twenty-four hours later the greater omentum was removed from each mouse, fixed in paraformaldehyde and stained immunohistochemically by the method described above. By using two-dimensional measurement software (Cosmozone I SA, Nikon, Japan), the number of BrdU-labelled cells per unit area was measured separately at milky spot sites and non-milky spot sites, which consist of serous membranes, adipose tissues and blood vessels. The counting procedure involved initially determining the total areas of the milky spot sites and the non-milky spot sites in each specimen, and then the number of the BrdU-labelled cells at each of the sites was measured separately. Finally the results were expressed as the number of BrdU-labelled cells per mm$^2$. The results were compared statistically by paired *t*-test.

### *Histological observation of B-16 PC melanoma cell infiltration*

The mouse B-16 PC melanoma cell line, which was established from the standard B-16 melanoma through serial intraperitoneal inoculation (24 times) in our laboratory and maintained by cell culture (RPMI-1640) *in vitro*, easily induces peritoneal dissemination by intraperitoneal inoculation. After harvesting by treatment with a 0.25% trypsin solution, B-16 PC melanoma cells at $10^6$ per mouse were inoculated intraperitoneally into ten mice (5-week-old male C57/BL; Shimizu Laboratory Animal Center). Two and 10 days after inoculation, five mice were sacrificed and the intraperitoneal distribution of the tumour cells were observed macroscopically and microscopically. We then examined the greater omentum using its stretch specimens stained with methyl green in order to distinguish black melanoma cells from the other cells.

### Results

### *Microscopic observation of P388 cell infiltration*

The greater omentum consisted of blood vessels, perivascular adipose tissue, serous membrane and milky spots. The milky spots were readily distinguished microscopically from other components of greater omentum, because milky spots exist along with blood vessels and consist of many aggregating cellular components such as macrophages, lymphocytes and mast cells (Dux, 1990), whereas the serous membranes consisted of a loose arrangement of mesothelial cells.

Figure 3 shows the microscopic views of the greater omenta which were taken from the mice inoculated i.p. with $10^6$ BrdU-labelled cells or $10^6$ control cells stained immunohistochemically using the anti-BrdU antibody. Figure 3a and b shows the greater omentum taken from mice that received $10^6$ BrdU-labelled cells. A large number of the BrdU-labelled cells stained brown selectively infiltrated the milky spots, which are the accumulation of green nuclei. Only a few labelled cells could occasionally be seen in the serous membranes. However, in the omenta taken from control mice, the labelled cells stained brown were seen at neither milky spots nor the other sites (Figure 3c and d).

### *Number of infiltrating P388 cells per unit area in tissue*

Since the BrdU-labelled cells were clearly distinguishable from the other cells at the milky spots and other structures in the greater omentum, they were easily counted even when a large number of cells were inoculated. The numbers of BrdU-labelled cells per unit area ($mm^2$) in the milky spot sites and the non-milky spot sites are shown in Table I. When $10^5$ cells were inoculated i.p., $15.8 \pm 13.3$ labelled cells $mm^{-2}$ (mean ± s.d., $n = 10$) were found in the milky spot sites and $9.14 \times 10^{-3} \pm 1.58 \times 10^{-2}$ cells $mm^{-2}$ were detected in the non-milky spot sites. Inoculation of $10^6$ cells produced $120 \pm 46.5$ labelled cells $mm^{-2}$ in the milky spot sites and $1.14 \times 10^{-1} \pm 7.82 \times 10^{-1}$ cells $mm^{-2}$ in the non-milky spot sites. In mice receiving $10^7$ cells, the numbers of labelled cells increased to $504 \pm 208$ cells $mm^{-2}$ in the milky spot sites and $7.07 \times 10^{-1} \pm 5.98 \times 10^{-1}$ in the non-milky spot sites. The ratios of the mean labelled cell number in the milky spot sites *vs* that in the non-milky spot sites were 1728:1, 1049:1, and 713:1 respectively. In all cases, there were statistically significant differences in the number of BrdU-labelled cells $mm^{-2}$ between milky spot sites and non-milky spot sites (paired *t*-test).

**Figure 3** Immunohistology of P388 leukaemia cells in the greater omentum of mice inoculated with $10^6$ BrdU-labelled cells or $10^6$ control cells (**a** and **c** × 20, **b** and **d** × 50). (**a** and **b**). Microscopic views of the greater omentum taken from mice that received $10^6$ BrdU-labelled cells. A large number of the BrdU-labelled cells stained brown selectively infiltrated the milky spots, which are the accumulation of green nuclei (large arrows). In contrast, a few labelled cells could occasionally be seen at serous membranes (small arrows). (**c** and **d**) Microscopic views of the greater omentum of mice inoculated with $10^6$ control cells. No labelled cells were seen at either milky spots or the other sites. (Large arrows show milky spots).

**Table I** Number of BrdU-labelled cells in milky spot and non-milky spot sites

| *Number of inoculated cell (cells)* | *Number of labelled cells in milky spot sites[a] (cells ± s.d. mm$^{-2}$)* | *Number of labelled cell in non-milky spot sites[a] (cells ± s.d. mm$^{-2}$)* | *Ratio[b]* | P-*value[c] (paired* t-*test)* |
|---|---|---|---|---|
| $10^5$ | 15.8 ± 13.3 | $9.14 \times 10^{-3} \pm 1.58 \times 10^{-2}$ | 1728:1 | <0.05 |
| $10^6$ | 120 ± 46.5 | $1.14 \times 10^{-1} \pm 7.82 \times 10^{-2}$ | 1049:1 | <0.01 |
| $10^7$ | 504 ± 208 | $7.07 \times 10^{-1} \pm 5.98 \times 10^{-1}$ | 713:1 | <0.01 |

[a]The number of infiltrating cells mm$^{-2}$ in milky spot or non-milky spot sites was calculated from ten specimens of greater omenta taken from ten mice inoculated with a different number of BrdU-labelled cells. [b]The given ratios were calculated from the mean number of the labelled cells in the milky spot sites *vs* that in the non-milky spot sites respectively. [c]Statistical significances were estimated from the number of BrdU-labelled cells between milky spot sites and non-milky spot sites using paired *t*-test.

*Histological observation of B-16 PC melanoma infiltration*

After observation of the mice sacrificed 2 days after inoculation of B-16 PC melanoma, we could find no macroscopic tumour intraperitoneally. However, microscopic study of the greater omentum showed many black melanoma cells infiltrating the milky spots. In contrast, in the other sites only a few melanoma cells could be seen (Figure 4a). In mice sacrificed 10 days after inoculation, we could detect some very small black tumour nodules macroscopically in the omentum, the mesentery and the gonadal fat. Microscopic observation of the omentum revealed that the black melanoma cells formed some large clusters at the milky spots and a few smaller ones at the non-milky spot sites (Figure 4b).

### Discussion

Milky spots are lymphoid tissue in the peritoneal cavity (Dux *et al.*, 1991) and are considered to be gates through which small particles are absorbed from the peritoneal cavity into the subperitoneum (Higgins and Bain, 1930). These milky spots are found mainly in greater omentum (Seifert, 1921), with relatively fewer of them appearing in the mesentery and the pelvic floor; no milky spots are found in other areas of the peritoneum (Hagiwara *et al.*, 1993). Milky spots may contribute to the peritoneal dissemination of cancer as sites of implantation. Some authors have reported that cancer cells infiltrate the milky spots in the early stages of peritoneal carcinomatosis in animal experiments (Dux, 1969; Green and Williams, 1978). The reason for this site-specificity of peritoneal carcinomatosis probably relates to the function of milky spots as gates through which small particles are transferred from the peritoneal cavity to the subperitoneum (Higgins and Bain, 1930). In addition, the cancer cells may readily adhere to the milky spots, since milky spots lack mesothelial cells (Beelen *et al.*, 1980).

However, those reports were based solely on subjective light or electron microscopic observations. No attempt has been made using human or animal experiments to quantify the numbers of viable cancer cells infiltrating the peritoneum at specific locations such as milky spots, because of the difficulties in determining the numbers of viable cancer cells infiltrating peritoneal tissues. In a previous report, we described the importance of greater omentum as a site of implantation of viable cancer cells in peritoneal dissemination, and also established a significant correlation between the number of cancer cells infiltrating peritoneal locations such as the greater omentum and the number of milky spots at those peritoneal locations by using a new experimental approach (Hagiwara *et al.*, 1993). In the present study, in order to corroborate these results, by labelling with bromodeoxyuridine (BrdU) and by immunohistochemical staining (Gratzner, 1982) we demonstrated that cancer cells selectively infiltrate milky spots. Furthermore, we quantitatively evaluated the site-specific implantation of cancer cells into milky spots by measuring the distribution of the labelled cells, especially in the omentum. In the omentum it was possible to compare the distribution of the BrdU-labelled cells in milky

**Figure 4** Histology of the greater omentum of mice inoculated i.p. with $10^6$ B-16 PC melanoma cells (**a** × 20, **b** × 5). (**a**) Microscopic view of the greater omentum taken from a mouse sacrificed 2 days after inoculation with B-16 PC melanoma cells. Many black melanoma cells selectively infiltrated the milky spots, which are the accumulation of green nuclei (large arrows). In contrast, only a few black melanoma cells can occasionally be seen at serous membranes (small arrows). (**b**) Microscopic view of the greater omentum of a mouse sacrificed 10 days after inoculation. At the milky spots, B-16 PC melanoma cells form large black nodules (large arrow). At the serous membrane site, a few smaller nodules of melanoma cells can be seen (small arrows).

spot and non-milky spot sites because omentum contains many milky spots and some other common components of peritoneal tissues such as serous membranes and adipose tissues.

We found immunohistochemically a large number of BrdU-labelled cells selectively infiltrating the milky spot sites after the inoculation of $10^6$ labelled cells. However, a few labelled cells were also detected at other sites in the omentum. In addition, the ratios of the mean labelled cell number in the milky spot sites *vs* that in the non-milky spot sites decreased as the numbers of inoculated cells increased. These results suggest that a small number of cancer cells floating in the peritoneal cavity tend to infiltrate the milky spots selectively, especially during the early stages of peritoneal carcinomatosis.

In the present study, we used P388 leukaemia cells as an experimental tumour, as in our previous experiments (Hagiwara *et al.*, 1993). This is because this cell line is a very useful experimental model of cancer metastasis to the regional lymph nodes or peritoneal cavity since there is a linear correlation between the survival time of mice and logarithm of the number of P388 cells inoculated into the peritoneum (Tsuruo *et al.*, 1980; Hagiwara *et al.*, 1993). Another reason is that these tumour cells are readily identified and counted microscopically after immunohistochemical staining since they rarely form clusters and are dispersed throughout the peritoneal cavity. In addition, the stretched omental preparation that we used in this study also has significant advantages over paraffin-embedded or frozen specimens. With stretch specimens it is possible to observe the whole greater omentum clearly and completely because of its transparency (Shimotsuma *et al.*, 1991; Dux *et al.*, 1993), but paraffin-embedded or frozen specimens provide a rather restricted view of the omentum. By using P388 leukaemia cells and immunohistochemical staining of the stretch specimens of the greater omentum, we could quantitatively analyse site-specific implantation of i.p. inoculated cancer cells to the milky spots in the omentum. Labelling with BrdU may alter the physiology of cancer cells. However, the procedure that we developed for labelling cells with BrdU at 5 $\mu g\ ml^{-1}$ for 24 h gave an adequate labelling index for immunohistochemical staining with the stretch specimens and was not detrimental to cell viability such that substantial infiltration of cancer cells into the peritoneal tissues occurred.

Finally, we examined intraperitoneally the distribution of the B-16 PC melanoma cell line after i.p. inoculation. Since this cell line contains intrinsic black melanin, it could easily be differentiated from other cells and observed its natural state without labelling and specific staining. Macroscopically, we found that the melanoma forms tumour clusters especially in the omentum, mesentery and gonadal fat, in which milky spots are distributed. Microscopically, we could demonstrate that the melanoma cells preferentially infiltrated milky spots in the omentum and became established there. This result leads to the conclusion that milky spots are very important implantation sites for peritoneal dissemination.

The results in this study suggest that cancer cells seeded i.p. specifically infiltrate milky spots during the initial stage of peritoneal metastasis.

### References

BEELEN RH, FLUITSMA DM AND HOEFSMIT ECM. (1980). The cellular composition of omentum milky spots and the ultrastructure of milky spot macropharge and reticulum cells. *J. Reticuloendothel. Soc.*, **28**, 585-599.

DUX K. (1969). Role of the greater omentum in the immunological response of mice and rats to the intraperitoneal inoculation of Ehrlich ascites tumor. *Arch. Immunol. Ther. Exp.*, **17**, 425-432.

DUX K. (1990). Anatomy of the greater omentum and lesser omentum in the mouse with some physiological implications. In *The Omentum. Research and Clinical Applications*, Goldsmith HS (ed.) pp. 19-43. Springer: New York.

DUX K, JANIK P AND SZANIAWASKA B. (1991). Kinetics of proliferation, cell differentiation, and IgM secretion in the omental lymphoid organ of B10 Sn mice following intraperitoneal immunization with sheep erythrocytes. *Cell Immunol.*, **32**, 97-109.

DUX K, SHIMOTSUMA M AND MAX WS. (1993). Technique for in situ excision distended samples of greater omentum from small laboratory animals. *Biotech. Histochem.*, **68**, 46-49.

GRATZNER HG. (1982). Monoclonal antibody to 5-bromo- and 5-iodo-deoxyuridine: a new reagent for detection of DNA replication. *Science*, **218**, 474-475.

GREEN JA AND WILLIAMS AE. (1978). The relationship between inflammatory responses and WBP1 tumor cell attachment to the rat omentum. *Eur. J. Cancer*, **14**, 1153-1155.

HAGIWARA A, TAKAHASHI T, SAWAI K, TANIGUCHI H, SHIMOTSUMA M, OKANO S, SAKAKURA C, TSUJIMOTO H, OSAKI K, SASAKI S AND SHIRASU M. (1993). Milky spots as the implantation site for malignant cells in peritoneal dissemination in mice. *Cancer Res.*, **53**, 687-692.

HIGGINS GM AND BAIN CG. (1930). The absorption and transference of particulate material by the great omentum. *Surg. Gynecol. Obstet.*, **50**, 851-860.

SEIFERT E. (1921). Zur Biologie des menschlichen grossen Netzes. *Arch. Klin. Circ.*, **116**, 510-517.

SHIMOTSUMA M, TAKAHASHI T, KAWATA M AND DUX K. (1991). Cellular subsets of the milky spots in the human greater omentum. *Cell Tissue Res.*, **264**, 599-601.

TSURUO T, NAGANUMA K, IIDA H AND TSUKAGOSHI S. (1980). Lymph node metastasis and effects of 1-β-D-arabinofuranosylcytosine, 5-fluorouracil, and their lipophilic derivatives in an experimental model system using P388 leukemia. *Cancer Res.*, **40**, 4758-4763.